# Spatio-temporal distribution of tubulin-binding cofactors and posttranslational modifications of tubulin in the cochlea of mice

**DOI:** 10.1007/s00418-020-01905-6

**Published:** 2020-07-25

**Authors:** Lukas Juergens, Linda Bieniussa, Johannes Voelker, Rudolf Hagen, Kristen Rak

**Affiliations:** 1 Department of Oto-Rhino-Laryngology, Plastic, Aesthetic and Reconstructive Head and Neck Surgery, The Comprehensive Hearing Center, University of Wuerzburg, Josef-Schneider-Strasse 11, 97080 Wuerzburg, Germany; 2 Department of Ophthalmology, University of Duesseldorf, Duesseldorf, Germany

**Keywords:** Tubulin-binding cofactors, Tubulin, Development, Cochlea, Posttranslational modifications, Hearing

## Abstract

The five tubulin-binding cofactors (TBC) are involved in tubulin synthesis and the formation of microtubules. Their importance is highlighted by various diseases and syndromes caused by dysfunction or mutation of these proteins. Posttranslational modifications (PTMs) of tubulin promote different characteristics, including stability-creating subpopulations of tubulin. Cell- and time-specific distribution of PTMs has only been investigated in the organ of Corti in gerbils. The aim of the presented study was to investigate the cell type-specific and time-specific expression patterns of TBC proteins and PTMs for the first time in murine cochleae over several developmental stages. For this, murine cochleae were investigated at the postnatal (P) age P1, P7 and P14 by immunofluorescence analysis. The investigations revealed several profound interspecies differences in the distribution of PTMs between gerbil and mouse. Furthermore, this is the first study to describe the spatio-temporal distribution of TBCs in any tissue ever showing a volatile pattern of expression. The expression analysis of TBC proteins and PTMs of tubulin reveals that these proteins play a role in the physiological development of the cochlea and might be essential for hearing.

**Electronic supplementary material:**

The online version of this article (10.1007/s00418-020-01905-6) contains supplementary material, which is available to authorized users.

## Introduction

The organ of Corti, located in the cochlea, is the receptor organ for hearing. Auditory signals are converted into action potentials that are transmitted to the auditory brainstem for further processing. The mature organ of Corti consists of various highly differentiated epithelial cells, which form during embryonic and postnatal development.

**Fig. 1:**
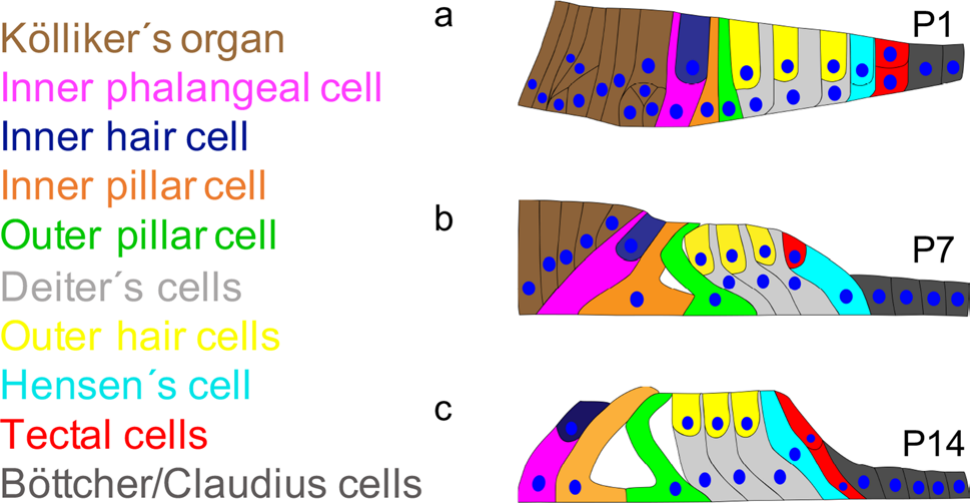
Postnatal development of the organ of Corti. The postnatal development is shown in this colour-coded illustration: brown = Kölliker’s organ, pink = inner phalangeal cell, dark blue = inner hair cell, orange = inner pillar cell, green = outer pillar cell, light grey = Deiters’ cells, yellow = outer hair cells, cyan = Hensen’s cells, red = tectal cells, dark grey = Bottcher and Claudius cells. **a** P1; In newborn mice, the organ of Corti presents itself as an epithelium of columnar cells. There are no extracellular spaces. **b** P7; The Corti tunnel initially opens basally between the inner and outer pillared cells. **c** P14; The Corti tunnel has reached its full size. During the entire development, the cells increase only slightly in height. Cells of Kölliker’s organ have gradually transformed into cuboidal cells of the inner sulcus and are no longer part of the cortical organ

During the embryonic phase, the future organ of Corti consists of an ectodermal tissue with four different precursor cells for neurons, hair cells, supporting cells and otic epithelium (Fritzsch et al. 2015). In newborn mice (P1), the organ of Corti presents itself as an epithelium of columnar cells without any extracellular spaces (Fig. 1a). Around P7 extracellular spaces develop (Fig. 1b) and at P14 the organ of Corti reaches its mature form (Fig. 1c) (Kikuchi and Hilding 1965). At this time, the sensory function of hearing has finally developed (Mikaelian and Ruben 1965). Different cells form a mosaic-like pattern during the development. Kölliker’s organ is the origin of the differentiation of sensory cells during the embryonic and early postnatal phase (Kelley 2007). Postnatally, Kölliker’s organ innervates the inner hair cells by depolarisation before the onset of hearing (Tritsch et al. 2007). After the onset of hearing, it transforms into cuboidal cells of the inner sulcus (Hinojosa 1977). The hair cells are the sensory cells in the cortical organ and are arranged in four rows: one row of inner hair cells and three rows of outer hair cells. The apical surface of the hair cells is lined with stereocilia, but only those of the outer hair cells have permanent contact with the tectorial membrane (Lim 1986). The supporting cells include the inner and outer pillar cell, the inner phalangeal cell, the Deiter’s cells (also called outer phalangeal cells), Hensen’s cells and the tectal cells. The Deiter’s cells and pillar cells contribute crucially to the stability of the sensory epithelium (Forge and Wright 2002; Zetes et al. 2012). Based on the presence of gap junctions, the supporting cells also play an important role in water and ion homeostasis of the perilymph and intercellular communication (Kikuchi et al. 2000). The inner pillar cell can be identified before all other cells of the Corti organ (E17 in rats) and is, therefore, believed to play an important role in the development of the Corti organ (Thelen et al. 2009). At the outer border of the organ of Corti are the Hensen’s cells and the tectal cells. They are thought to play a role in potassium recycling (Wangemann 2002), to actively participate in protection against trauma from high-intensity sound exposure (Flock et al. 1999) and to have the ability to differentiate into Deiters’ and hair cells (Malgrange et al. 2002) (Fig. 1).

Microtubules (MT) consisting of alpha- and beta-heterodimers form an essential part of the cytoskeleton, and play an important role in maintaining cellular structure, intracellular transport and mitosis. They represent a heterogenous group consisting of different building blocks (tubulin isotypes) and modifications (post-translational modifications) to perform these diverse functions (Gadadhar et al. 2017). During tubulin synthesis, five tubulin-specific chaperone proteins known as tubulin-binding cofactors (TBCA—TBCE) are involved in forming alpha–beta-heterodimers from newly synthesised alpha- and beta-tubulin monomers (Tian et al. 1996; Cowan and Lewis 2001; Szymanski 2002). There are many different opinions about the interaction of these chaperones (Francis et al. 2017). The important role of the TBC proteins is underlined by various diseases and syndromes caused by the dysfunction or mutation of these proteins (Bartolini et al. 2002; Parvari et al. 2002; Wang et al. 2006; Zhang et al. 2013). Furthermore, it was found that a missense mutation of the TBCE gene leads to progressive motor neuropathy and hearing loss in mice (Bommel et al. 2002; Volkenstein et al. 2009). The latter is caused by selective apoptosis of the outer hair cells of the organ of Corti and a disturbed MT distribution of the auditory nerve fibres (Rak et al. 2013).

Post-translational modifications (PTMs) of tubulin enable the creation of various subpopulations of tubulin by promoting different characteristics (Westermann and Weber 2003; Verhey and Gaertig 2007; Hammond et al. 2008). These include positive and negative effects on the stability of microtubules (Black et al. 1989; Peris et al. 2009; Lacroix et al. 2010; Sirajuddin et al. 2014; Aillaud et al. 2016). Tannenbaum and Slepecky (1997) found a cell- and time-specific distribution of PTMs in the organ of Corti in gerbils. Later, Hallworth and Luduena (2000) and Jensen-Smith et al. (2003) mapped the distribution of beta-tubulin isotypes in the organ of Corti in gerbils during the first postnatal month. Three different isotypes were found in the organ of Corti. While all three isotopes are expressed in all cell types at P0, cell-type-specific reductions occur which lead to different isotype combinations in each cell type.

These findings provide insight into the complex intracellular changes in MT distribution and function occurring during early postnatal development.

To further investigate these mechanisms, two objectives were set for this study in which developing murine cochleae were investigated by immunofluorescence analysis. The first objective was to examine cell type-specific and time-specific expression patterns of TBC proteins. The second objective was to determine whether there is a spatio-temporal change in the distribution of post-translational modifications of tubulin. The results of these investigations will lead to a deeper understanding of the specific development of cochlear cells and possibly help to clarify why different cells show their distinct cellular morphology, which may be linked to altered stability due to differences in the MT distribution.

## Materials and methods

### Preparation of tissue

Naval Medical Research Institute (NMRI) mice at P1, P7 and P14 were sacrificed by cervical dislocation, decapitated and cochleae were dissected. For sections, 4% paraformaldehyde in 0.1 M sodium phosphate buffer (PFA) was carefully instilled via the round and oval window. Cochleae were then incubated in 4% PFA for 2 h at 4 °C. For sections of P14 the cochleae were further decalcified in 0.135 M ethylenediaminetetraacetic acid (EDTA) at room temperature for 3 h and stored in 0.1% sodium citrate buffer at 4 °C. Cochleae were then incubated with 30% saccharose overnight for equilibration, cryo-protected Tissue-Tek O.C.T. (Sakura) and stored at − 80 °C. For whole-mount preparations of P14 animals, cochleae were incubated in 4% PFA for 2 h at 4 °C, without penetrating the oval or round window.

### Immunohistochemistry of sections

Sections of 10 μm were cut by cryostat (Leica). For immunofluorescence analysis, sections were post-fixed in 4% PFA for 10 min, rinsed in 0.05 M phosphate-buffered saline three times and blocked in 10% bovine serum albumin (BSA) in 0.1% Triton X-100 for 30 min. Primary antibodies were incubated in 1% BSA in 0.1% Triton X-100 overnight at 4 °C at the following concentrations: rabbit polyclonal against TBCA (1:50), TBCB (1:100), TBCC (1:200), TBCD (1:100) and TBCE (1:100, all Proteintech), mouse monoclonal against tyrosinated tubulin (1:500, Millipore Merck), rabbit polyclonal against detyrosinated tubulin (1:500, Millipore Merck), mouse monoclonal against acetylated tubulin (1:500, Sigma-Aldrich) or mouse monoclonal against polyglutamylated tubulin (1:500, AdipoGen). Sections were then rinsed three times with tris-buffered saline with Tween20 (TBS-T) and incubated for one hour in 1% BSA in TBS-T with secondary antibody coupled to Alexa 488, Alexa A555 (both either goat anti-rabbit goat anti-mouse, 1:500, Invitrogen) and DAPI (1:1000, Sigma-Aldrich). After washing three times with TBS-T sections were embedded in Mowiol.

### Immunohistochemistry of whole mounts

For whole mounts, cochleae were dissected as previously described (Montgomery and Cox 2016). For immunofluorescence analysis, the tissue was blocked in 10% BSA in 0.1% Triton X-100 for 1 h. Primary antibodies were incubated in 1% BSA in 0.1% Triton X-100 overnight at 4 °C at the following concentrations: mouse monoclonal against β-tubulin (1:500, Sigma- Aldrich), rabbit polyclonal against TBCA (1:50), TBCB (1:100), TBCC (1:200), TBCD (1:100) and TBCE (1:100, all Proteintech). Subsequently, the whole mounts were then rinsed three times with TBS-T and incubated for one hour in 1% BSA in TBS-T with secondary antibody coupled to Alexa Fluor 488, Alexa Fluor A555 (both either goat anti-rabbit goat anti-mouse, 1:500, Invitrogen), DAPI (1:1000, Sigma-Aldrich) and Alexa Fluor 488 Phalloidin (1:250, Invitrogen). After washing three times with TBS-T the whole mounts were embedded in Mowiol.

### Image acquisition

Image acquisition was performed with a IX81 microscope combined with a FV1000 confocal laser scanning system, a FVD10 SPD spectral detector and diode lasers of 405, 473 and 555 nm (Olympus). Objectives used were UPLSAPO 40x (oil, numerical aperture, NA, 1.35) or UPLSAPO 20x (air, numerical aperture, NA, 0.75) (Olympus). For P1, P7 and P14 sections, images were acquired directly. For P14 whole-mounts stacks were created in 0.5 µm steps in baso-apical direction. Finally, the stacks were reconstructed orthogonally in the XZ plane in ImageJ.

### Western-blot

Proteins were extracted out of Hela cells using lysis buffer (1% Nonidet P40, 50 mM Tris–HCl pH 7.5, 150 mM NaCl, 10% Glycerol, 100 mM NaF, 100 mM Na-pyrophsophate, 200 mM Na-ortho-vanadate, 0.5 M EDTA pH 8.0). For homogenizing, the suspension was sonified with a Hielscher Sonifier (UP50H, Sonotrode M1) by 3 cycles á 0.5 s at 80% power. To quantify the protein concentration of the cell lysates, Pierce BCA Assay Kit was used according to the kit protocol and analysed with an Eppendorf Spectrophotometer. Western Blotting was performed by materials of ThermoScientific. For the analyses, 20 µg of protein extract was diluted to a total volume of 30 µl with 10xNuPage Sample Reducing Agent containing 500 mM dithiothreitol (DTT) and incubated at 82 °C for 2 min. Samples were separated electrophoretically in Novex Wedge Well 4–20% Tris–Glycine gels with colorimetric PageRuler Protein ladder (#26616) and blotted with a Power Blotter Station (PB0010) on related Select Transfer Stacks including nitrocellulose membrane (PB3310). Blocking and antibody incubation was performed in heat-inactivated and filtered 1xTBST including 5% blotting grade milk powder (Roth, T145.1) overnight at 4 °C. For detection, optimal concentrations for all antibodies were evaluated. Post-translational modifications antibodies were used at 1:5000 and tubulin-binding cofactors antibodies at 1:500. As loading control either β-tubulin (1:5000, Sigma) or GAPDH (1:2500, SySy) antibodies were applied. Protein bands were visualized using horseradish peroxidase (HRP)-coupled secondary antibodies and Pierce ECL Western Blotting Substrate (#32209) as substrate. Image acquisition was done using ChemiDoc MP Imaging System (Biorad).

## Results and discussion

In the present study, the distribution of TBC proteins and PTMs of tubulin are described for the first time in the postnatal developing murine cochlea. The timespan from P1 until P14 was chosen because; (1) the expression of microtubules starts at P1 in the organ of Corti (Hallworth et al. 2000), (2) the organ of Corti reaches its mature form at P14 (Kikuchi and Hilding 1965) and (3) the hearing of the mice starts at P10 reaching adult sensitivity by P14 (Mikaelian and Ruben 1965). Consecutively, the timespan with the possible greatest changes in expression changes is located from P1 until P14.

Different techniques were applied for the investigations. Cochleae of P1 and P7 mice were processed directly for sectioning and staining. The cochleae of P14 mice had to be decalcified before further processing. This resulted in difficulties of antibody binding of all TBC antibodies, similar which has been described before after decalcification (Soliman 1988; Ramos-Vara and Miller 2014). To solve this problem cochlea of P14 mice were further processed without decalcification as whole-mount preparations (Montgomery and Cox 2016). To show similar pictures of the cochlea from all ages, pictures of the whole-mount preparations were taken by confocal microscope and stacks reconstructed orthogonally for presentation. In addition, experiments with only primary or secondary antibodies were performed, which showed only little background staining (Suppl. Fig. 1).

### Antibody validation

For antibody validation, Western-blot analysis was performed (Suppl. Fig. 2). Post-translational modifications displayed a molecular weight in the range 55 kDa except detyrosinated tubulin, which molecular weight is detected at 40 kDa. The loading control band of GAPDH was at 36 kDa (Suppl. Fig. 2a). Western blotting of tubulin-binding cofactors TBCA antibody detected a molecular weight of about 13 kDA, TBCB of 27 kDa, TBCC of 39 kDa, TBCD of 120 kDa, TBCE of about 70 kDa and the loading control of β-tubulin antibody of 50 kDa (Suppl. Fig. 2b).

### Volatile expression of TBC proteins during the postnatal development of the organ of Corti

#### P1

**Fig. 2:**
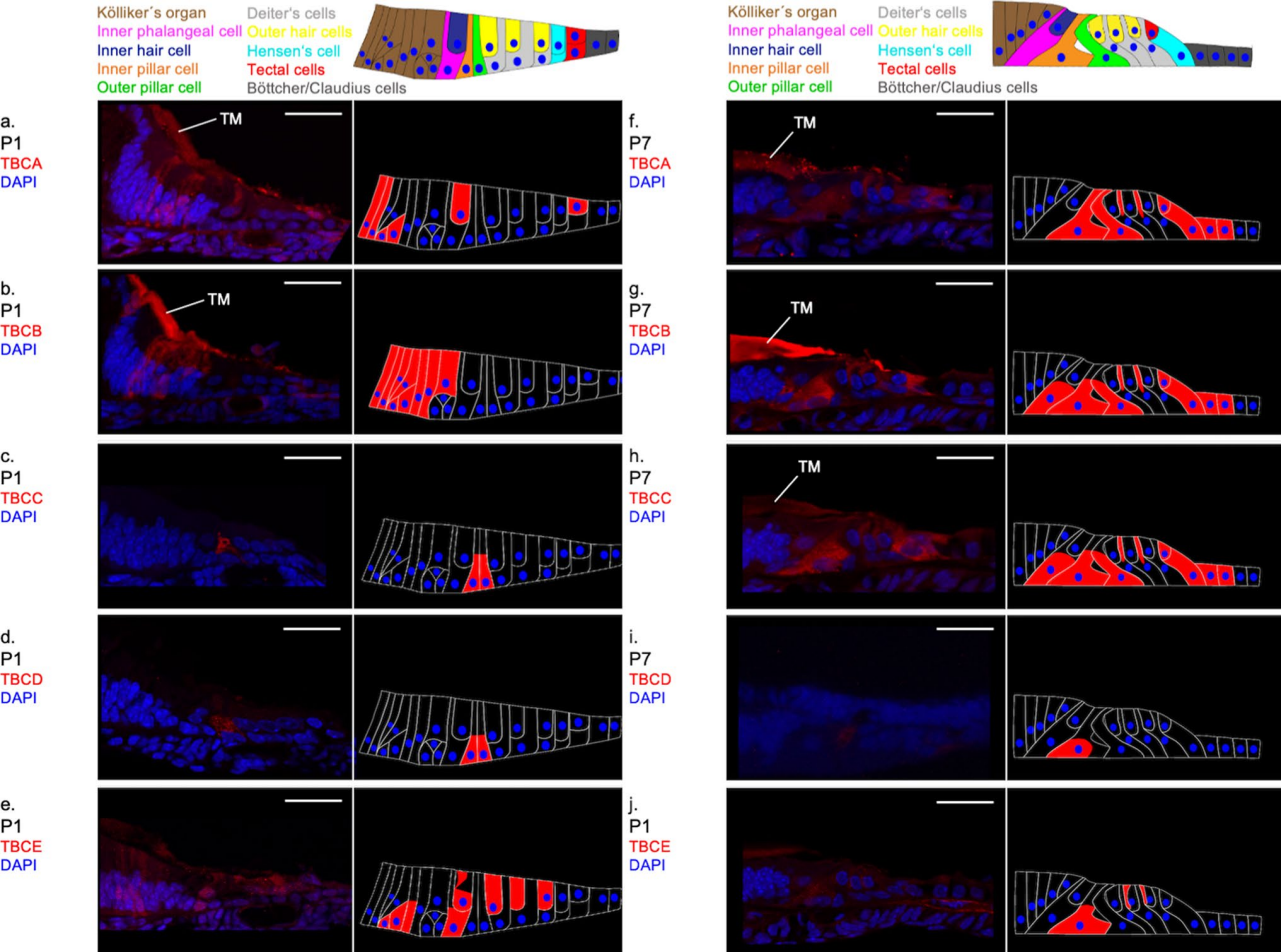
Distribution of TBC proteins in the organ of Corti at P1 and P7. **a**–**e** age P1; **a** TBCA expression is found in the inner area of Kölliker’s organ, the inner hair cell and a tectal cell. **b** TBCB is expressed in the cells of the Kölliker’s organ. **c** TBCC labelling is detected in the basal half of the two pillar cells. **d** Similar TBCD is expressed in the pillar cells. **e** TBCE staining is found in the basal cells of Kölliker’s organ and the basal half of the inner phalangeal cell. Diffuse staining is visible around the nucleus and below the apical surface of the inner hair cells. The three outer hair cells are also marked. **f**–**j** age P7: **f** TBCA is detected in the inner and outer pillar cells, phalangeal extensions of the three Deiters’ cells, tectal cells and Hensen’s cells. **g** Similar to TBCA, TBCB is detected in the inner and outer pillar cells, the three Deiters’ cells, the tectal cells and Hensen’s cells. **h** The expression of TBCC is found in the supporting cells (inner and outer pillar cells, the phalangeal extensions of the three Deiters’ cells, the tectal cells and Hensen’s cells). **i** TBCD staining is found in the basal parts of the inner pillar cell. **j** In addition to the inner pillar cells, TBCE is also detected in the and the basal half of the inner phalangeal cell phalangeal extensions of Deiters’ cells. (*TM* tectorial membrane with unspecific staining) (Scale bar = 25 µm)

At the developmental stage P1 (Fig. 1a), the TBCA antibody shows a staining of cells in the inner area of Kölliker’s organ as well as the inner hair cell and a tectal cell (Fig. 2a). Similar to TBCA, the antibody TBCB marks cells of Kölliker’s organ (Fig. 2b). The expression of TBCC is different from TBCA and TBCB with extensive antibody labeling in the basal half of the two pillar cells (Fig. 2c). Like TBCC, TBCD staining is found in the basal half of the two pillar cells at P1 (Fig. 2d). In contrast, TBCE staining is found in the basal cells of Kölliker’s organ and the basal half of the inner phalangeal cell. Diffuse staining is visible around the nucleus and below the apical surface of the inner hair cells. Additionally, the TBCE antibody stains the three outer hair cells (Fig. 2e).

#### P7

At P7 (Fig. 1b), Köllliker’s organ and inner hair cells, however, show no expression of TBCA anymore. The antibody, however, stains the entire inner and outer pillar cell, the phalangeal extensions of the three Deiters’ cells, tectal cells and Hensen’s cells (Fig. 2f). Cells of the organ of Corti showed first expression of TBCB at P7. The pattern resembles that of TBCA and TBCC: the antibody is expressed in the basal half of the inner phalangeal cell as well as the inner and outer pillar cells (Fig. 2g). The TBCC antibody stains the basal half of the inner phalangeal cell, the inner and outer pillar cells, phalangeal extensions of the three Deiters’, tectal and Hensen’s cells (Fig. 2h). TBCD antibody shows a very different expression pattern than TBCA, TBCB and TBCC: only the inner pillar cells in the region of the cell nucleus are stained (Fig. 2i). In addition, TBCE antibody marks the basal half of the inner phalangeal cell and the phalangeal extensions of the Deiters’ cells (Fig. 2j).

#### P14

**Fig. 3:**
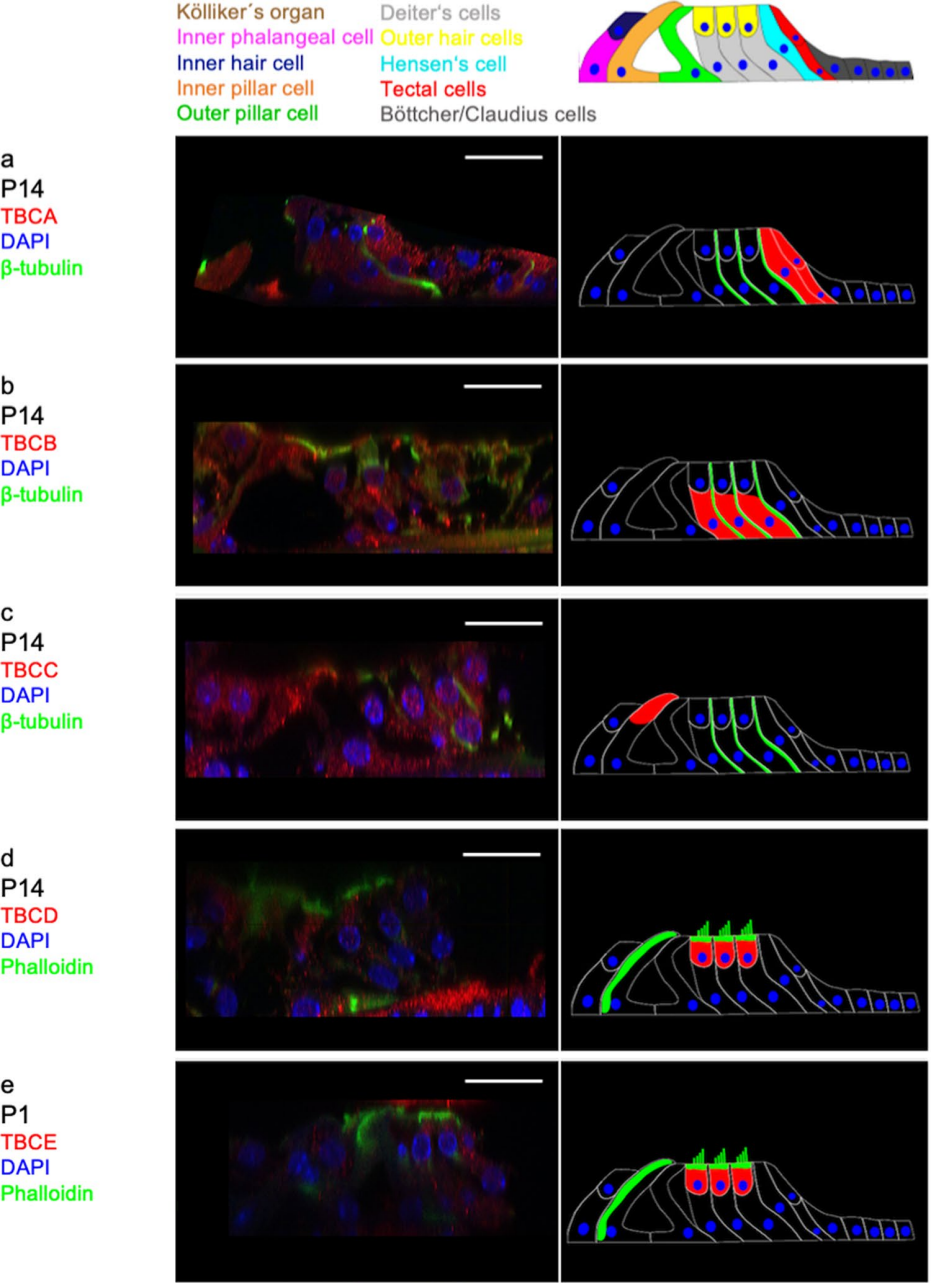
Distribution of TBC proteins in the organ of Corti at P14. **a** TBCA labelling is found in both the tectal and Hensen’s cells. β-Tubulin stains the phalangeal extension of the Deiters’ cells. **b** TBCB expression is detected in basal cell half of the Deiters’ cells, the phalangeal processes are stained by β-tubulin. **c** The expression of TBCC is found in the apical part of the inner pillar cell, β-tubulin in the Deiters’ cells. **d** Staining of TBCD is visible in the cell body of outer hair cells. Phalloidin stains the cuticular plate and stereocilia of the outer hair cells and the inner pillar cell. **e** TBCE is detected in the outer hair cells, whereas Phalloidion marks the cuticular plate and the stereocilia of the outer hair cells and a bundle-like structure extending from the apex to the base of inner pillar cell. (Scale bar = 25 µm)

At this developmental stage (Fig. 1c), TBCA labelling is found in both the tectal and Hensen’s cells (Fig. 3a). From P7 to P14 the expression pattern of TBCB changes completely: The phalangeal extensions of the three Deiters’, tectal and Hensen’s cells are now marked. In addition, microtubule bundles extending from the basal cell wall to the outer hair cell in the basal cell half of the Deiters’ cells are also stained by TBCB (Fig. 3b). At P14, TBCC expression is only detected in the apical part of the inner pillar cell (Fig. 3c). In the Deiters’ cells, bundle-like beta-tubulin labelled structures extend from the basal cell pole along with the phalangeal extensions to the apical surface (Fig. 3a–c). Diffuse TBCD labelling is found in the outer hair cells, whose stereocilia show phalloidin staining (Fig. 3d). In addition, TBCE staining can be detected in the outer hair cells whose stereocilia show phalloidin staining at P14 (Fig. 3d, e).

Discussing these results, it has to be acknowledged, that at P1 only TBCC and TBCD share a similar location (basal half of the pillar cells) while TBCA, TBCB and TBCE are expressed in various cells of the organ of Corti. A far more homogenous pattern is found at P7: TBCA, TBCB and TBCC are detectable in nearly all cell types and share the same intracellular localization. However, no TBCA, TBCB or TBCE can be found in any of the cells in which there was an expression at P1. There is a reduction in the pattern of expression from P7 to P14. All TBC proteins are traceable in one single cell type only—some in cell types or intracellular localizations where there was no previous expression at P1 and P7. Since this is the first time that the expression of TBC proteins has been studied in any tissue, no comparisons with other studies can be made. The knowledge of these proteins is still quite limited and studies have so far only been carried out in-vitro. Thus, no reliable cell-type-specific function can be deduced. It is assumed that the existence of all five chaperones is necessary for tubulin synthesis (Tian et al. 1996; Francis et al. 2017) and that disturbing their equilibrium results in loss of microtubules (Bhamidipati et al. 2000; Nolasco et al. 2005; Francis et al. 2017). This is also shown by animal models with a mutation in TBC proteins: A missense mutation of the TBCE gene causes selective apoptosis of the outer hair cells and faulty MT distribution of the auditory nerve fibers a progressive motor neuropathy and hearing loss in mice (Rak et al. 2013). In addition, a disease with altered TBC proteins are also found in humans: Mutation of the retinitis pigmentosa 2 protein (RP2) which not only has a sequence similar to TBCC but also a functional overlap in vivo, leads to severe form of X-linked retinitis pigmentosa (Bartolini et al. 2002; Grayson et al. 2002).

### Distribution patterns of posttranslational modifications of tubulin during postnatal cochlear development

#### P1

**Fig. 4:**
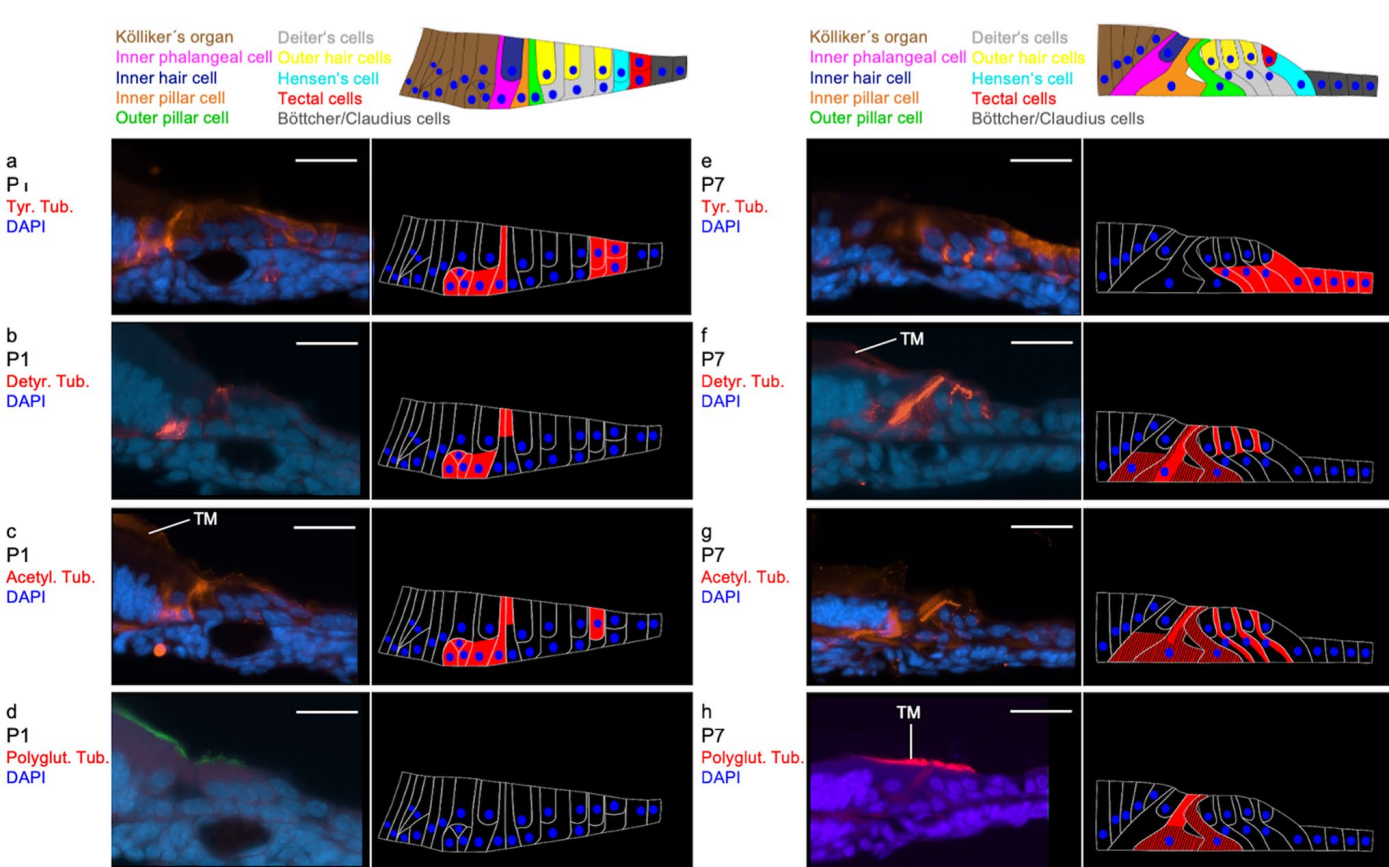
Distribution of tubulin PTMs in the organ of Corti at P1 and P7. **a**–**d**: age P1; **a** Tyrosinated tubulin expression is found in the basal cells of Kölliker’s organ, in the inner pillar cells as well as in the tectal and Hensen’s cells. **b** Detyrosinated is detected in basal sections of Kölliker’s organ as well as in parts of the inner and outer pillar cell. **c** acetylated tubulin marks the basal sections of Kölliker’s organ, parts of the inner and outer pillar cell and a boundary cell. **d** There is no antibody marking of polyglutamylated tubulin in the organ of Corti or adjacent cells. (Scale bar = 25 µm). **e**–**h** age P7; **e** Tyrosinated tubulin staining is found in the basal half of the Deiters’, tectal and Hensen’s cells. **f** Expression of detyrosinated tubulin is detected in the basal half of the inner phalangeal cell and the inner pillar cell. The outer pillar cell also stains diffusely. Bundle-like structures in parts of the Deiters’ cells and a dense structure extending through the entire inner pillar cell are also intensively stained. **g** Acetylated tubulin marks the inner phalangeal cell and the inner pillar cell strongly, the outer pillar cell diffusely and bundle like structures in the Deiters’ cells. **h** Weak polyglutamylated tubulin staining of the basal half of both pillar cells as well as a more intensive polyglutamylated tubulin staining of the apical half of the inner pillar cell is visible. (*TM* tectorial membrane with unspecific staining) (Scale bar = 25 µm)

At developmental day P1 (Fig. 1a) tyrosinated, detyrosinated and acetylated tubulin are found in the basal cells of Kölliker’s organ and in the pillar cells (Fig. 4a–c). Furthermore, tyrosinated and acetylated tubulin can be detected in the border cells (tectal and Hensen’s cells). Polyglutamylated tubulin, however, cannot be found in any cell of the organ of Corti (Fig. 4d). The former three antibodies are particularly expressed in the apical parts of the pillar cells. This resembles the pattern detected by Tannenbaum and Slepecky (1997) in gerbils. The apex of the pillar cells hosts the microtubule organising center (MTOC) from which new microtubules elongate (Tucker et al. 1992, 1995). Furthermore, these three PTMs are expressed in the area around the basal part of Kölliker’s organ and the habenula perforata. It is assumed that intrinsic, spontaneous activity is generated in the former which promotes the development of primary afferent nerve fibers (Tritsch et al. 2007). The latter is a perforated section of the basilar membrane through whose holes the nerve fibers run from the organ of Corti towards the modiolus and vice versa (Raphael and Altschuler 2003). Ito et al. (1995) were able to detect beta-tubulin in this area of the organ of Corti. While it is not possible from our own immunohistochemical stainings to differentiate whether the PTMs are expressed in Kölliker’s organ or within the nerves running through the habenula perforata, the images of Tannenbaum and Slepecky (1997) display an expression of PTMs in the nerves.

#### P7

On the seventh postnatal day (P7; Fig. 1b), detyrosinated, acetylated and polyglutamylated tubulin show similar expression patterns: First, the three tubulin modifications are expressed in both pillar cells (Fig. 4f–h). While detyrosinated microtubules extend as thick strand-like structures from the cell apex to the cell base, acetylated tubulin is detectable in the apical part of the cell and polyglutamylated tubulin ubiquitously. Detyrosinated and acetylated tubulin are also present in the Deiters’ cells as strand-like bundles. This expression pattern correlates with the distribution described by Tannenbaum and Slepecky (1997) in gerbils.

In contrast to the other three posttranslational modifications, tyrosinated tubulin shows no expression in the pillar cells, but the strong expression in the basal half of the Deiters’ cells and diffuse expression in the tectal and Hensen’s cells (Fig. 4e). Specific functions and properties of these cells at this stage of development are not yet known. Herein lies one of the main differences in the comparison of the present work with the publication of Tannenbaum and Slepecky (1997): In gerbils, tyrosinated tubulin could be detected, above all, in the base of the inner pillar cell, in the entire outer pillar cell and in the phalanges of the Deiters’ cells. Similarly to the expression of beta-tubulin (Ito et al. 1995), in the first postnatal week there is a reduction in the PTMs within the nerve fibres above the habenula perforata.

#### P14

**Fig. 5:**
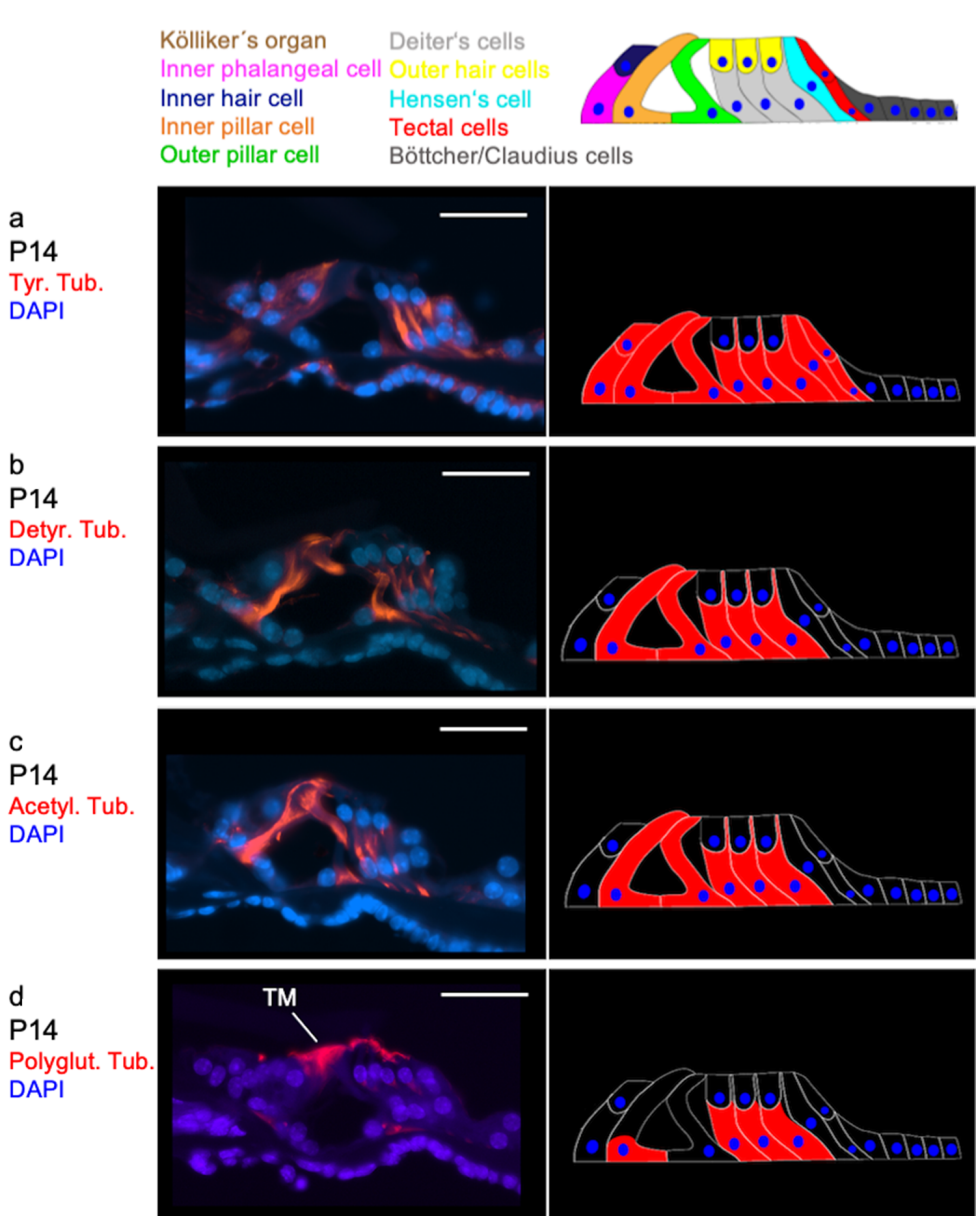
Distribution of tubulin PTMs in the organ of Corti at P14. **a** Faint labelling of tyrosinated tubulin is found in the inner hair cell, the inner phalangeal cells, tectal and Hensen’s cells as well as in the inner and outer pillar cells. Furthermore, a dense staining reaching from the base to the phalangeal processes in all three Deiters’ cells. **b** Detyrosinated tubulin antibodies mark elongated structures in the inner and outer pillar cells as well as in the three Deiters’ cells. In the pillar cells, the bundle-like structures extend from the basal cell pole to the head plate, and in the Deiters’ cells from the basal cell pole to the phalanges. **c** Acetylated tubulin staining is found in the inner and outer pillar cells and bundle-like structures in the Deiters’ cells. **d** Polyglutamylated tubulin is found in oblong structures in the three Deiters’ cells, and somewhat weaker in the inner pillar cell. The bundle-like structures in the pillar cells extend from the basal cell pole to the head plate and in the Deiters’ cells from the basal cell pole to the beginning of the phalangeal processes. Circumscribed antibody marking is found in the apical part of the inner hair cell. (*TM* tectorial membrane with unspecific staining) (Scale bar = 25 µm)

At P14 (Fig. 1c), tyrosinated, detyrosinated, acetylated and polyglutamylated tubulin are expressed in all supporting cells (Fig. 5a–d). The antibodies of the first three show intensively stained, elongated structures in the three Deiters’ cells. In the pillar cells, bundle-like structures extend from the basal cell pole to the head plate, and in the Deiters’ cells from the basal cell pole to the phalanges. Tyrosinated tubulin is also more weakly expressed in the inner hair cell, the inner phalangeal cells, tectal and Hensen’s cells.

Expression of polyglutamylated tubulin is detected in the basal parts of the inner pillar cell and the Deiters’ cells. Circumscribed antibody marking is found in the apical part of the inner hair cell. This contradicts the findings of Tannenbaum and Slepecky (1997) who found tyrosinated tubulin not arranged in microtubule bundles but is present ubiquitously in the cells of the organ of Corti. Furthermore, expression of polyglutamylated tubulin is detected along with the entire microtubule bundle of the pillar and Deiters’ cells in gerbils.

In the presented study, PTMs were detected in the supporting cells and lateral cells (i.e. tectal and Hensen’s cells). This seems reasonable since pillar and Deiters’ cells feature prominent microtubule bundles (Tucker et al. 1998), while there are only a few microtubules present in the hair cells (Renauld et al. 2015). During maturation, the microtubules develop from the cell apex to the basal part of the cells. This is explained by the location of MTOC (Tucker et al. 1992, 1998; Henderson et al. 1994, 1995).

Species-specific differences are one possible explanation for the described differences. Other studies on PTMs and tubulin isotypes have hitherto only been performed in gerbils. The structure of the outer hair cells exhibits species-specific differences: For example, in mice there is only one layer of subsurface cisternae (SSC), a component of the lateral wall of the outer hair cells, while in gerbils there are 2–7 layers (Raphael and Altschuler 2003). The SSC are connected to a microtubule network (Flock et al. 1986; Raphael and Wroblewski 1986; Raphael et al. 1993). The function of the SSC is still largely unclear. They have a similar structure to the Golgi apparatus and the endoplasmic reticulum (Pollice and Brownell 1993) which play a central role in protein biosynthesis as well as endo- and exocytosis. In addition, cross-species differences in the structure of the cytoskeleton were observed: In contrast to monkeys, rats and mice, the outer hair cells in guinea pigs have a network of actin and spectrin below the cuticular plate (Raphael et al. 1994).

However, there could also be a more technical explanation for the differences. Tannenbaum and Slepecky (1997) used different antibodies. Their antibody against tyrosinated tubulin was immunised in mice, while the one that was used in this study was immunised in rats. The later has been described in a methodical overview of PTMs antibodies (Magiera and Janke 2013). According to the manufacturer’s information, both antibodies bind to different epitopes.

## Conclusion

In the present study, the distribution of TBC proteins and PTMs of tubulin are described for the first time in the developing murine cochlea. The spatio-temporal development of TBC proteins shows a volatile pattern from which no cell type-specific function can be deduced. While there are parallels in the spatio-temporal development of PTMs in gerbils and mice, there are also quite profound differences.

## Electronic supplementary material

Below is the link to the electronic supplementary material.

Supplementary file1 Supplementary Figure 1 Control incubations of antibodies and test of background staining. For control incubation tests, stainings were performed, in which the secondary antibody was omitted. No unspecific staining could be detected (**a**–**i**). In addition, the background staining of the secondary antibodies war tested. Only faint background staining of Alexa 555 antibody could be detected (**j**). (Scale bar = 25 µm) (TIFF 5584 kb)

Supplementary file2 Supplementary Figure 2 Antibody validation by Western-blot analysis. Whole-cell lysate of HeLa cell culture was analysed on western blot probed with antibodies against polyglutaminated, acetylated, detyrosinated and tyrosinated PTMs of tubulin and GAPDH as reference protein (**a**). Antibodies against TBC proteins were investigated and analysed (loading control β-tubulin) (**b**) (TIF 2841 kb)
