# Supplementary figures and images for: Spatio-temporal distribution of tubulin-binding cofactors and posttranslational modifications of tubulin in the cochlea of mice
Source: Histochem Cell Biol. 2020 Jul 25;154(6):671–81. doi: 10.1007/s00418-020-01905-6 (PMC7723944; doi:10.1007/s00418-020-01905-6)

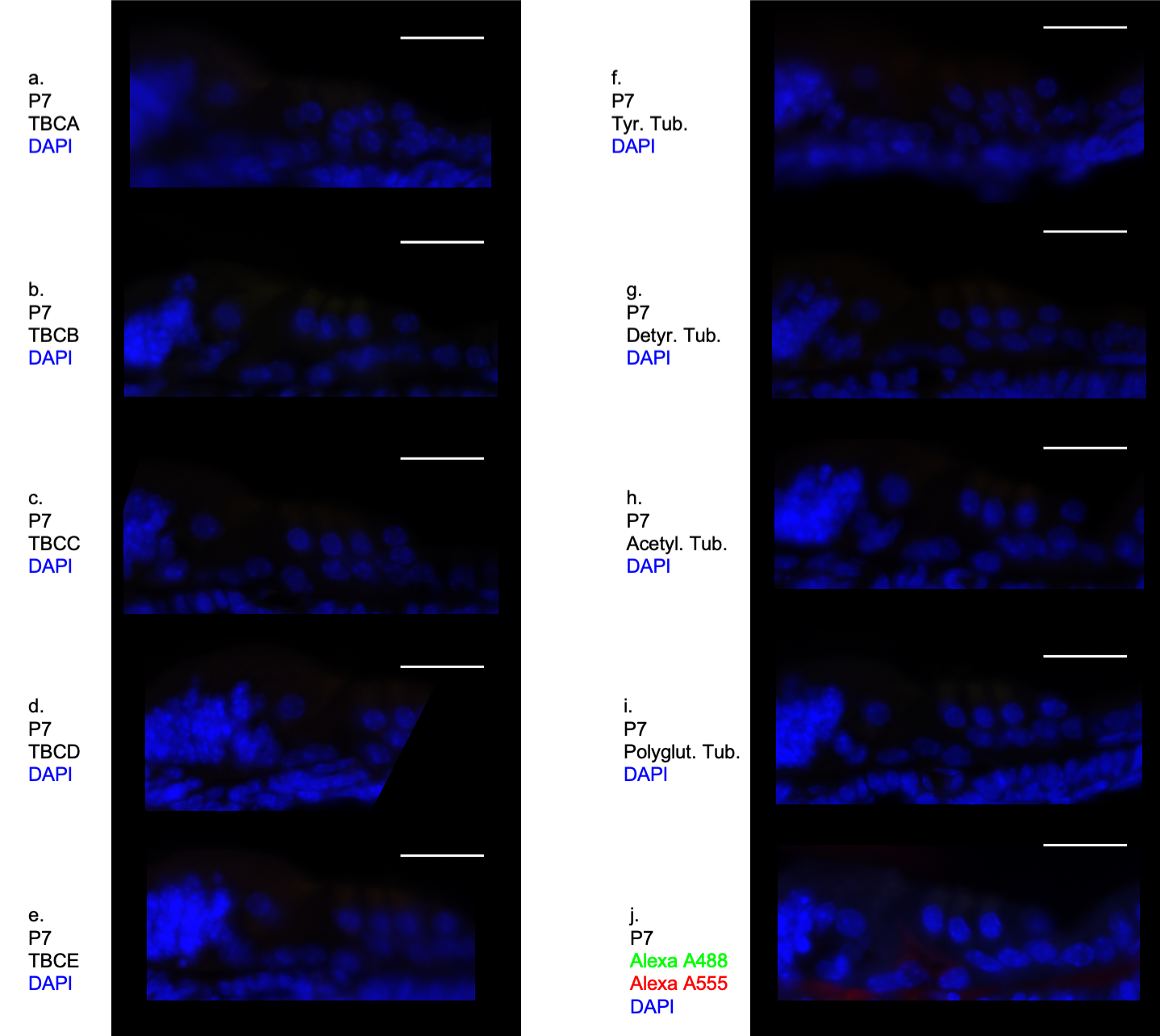

Supplement: Supplementary file 1 — Supplementary file1 Supplementary Figure 1 Control incubations of antibodies and test of background staining. For control incubation tests, stainings were performed, in which the secondary antibody was omitted. No unspecific staining could be detected (a–i). In addition, the background staining of the secondary antibodies war tested. Only faint background staining of Alexa 555 antibody could be detected (j). (Scale bar = 25 µm) (TIFF 5584 kb) [file 418_2020_1905_MOESM1_ESM.tiff]

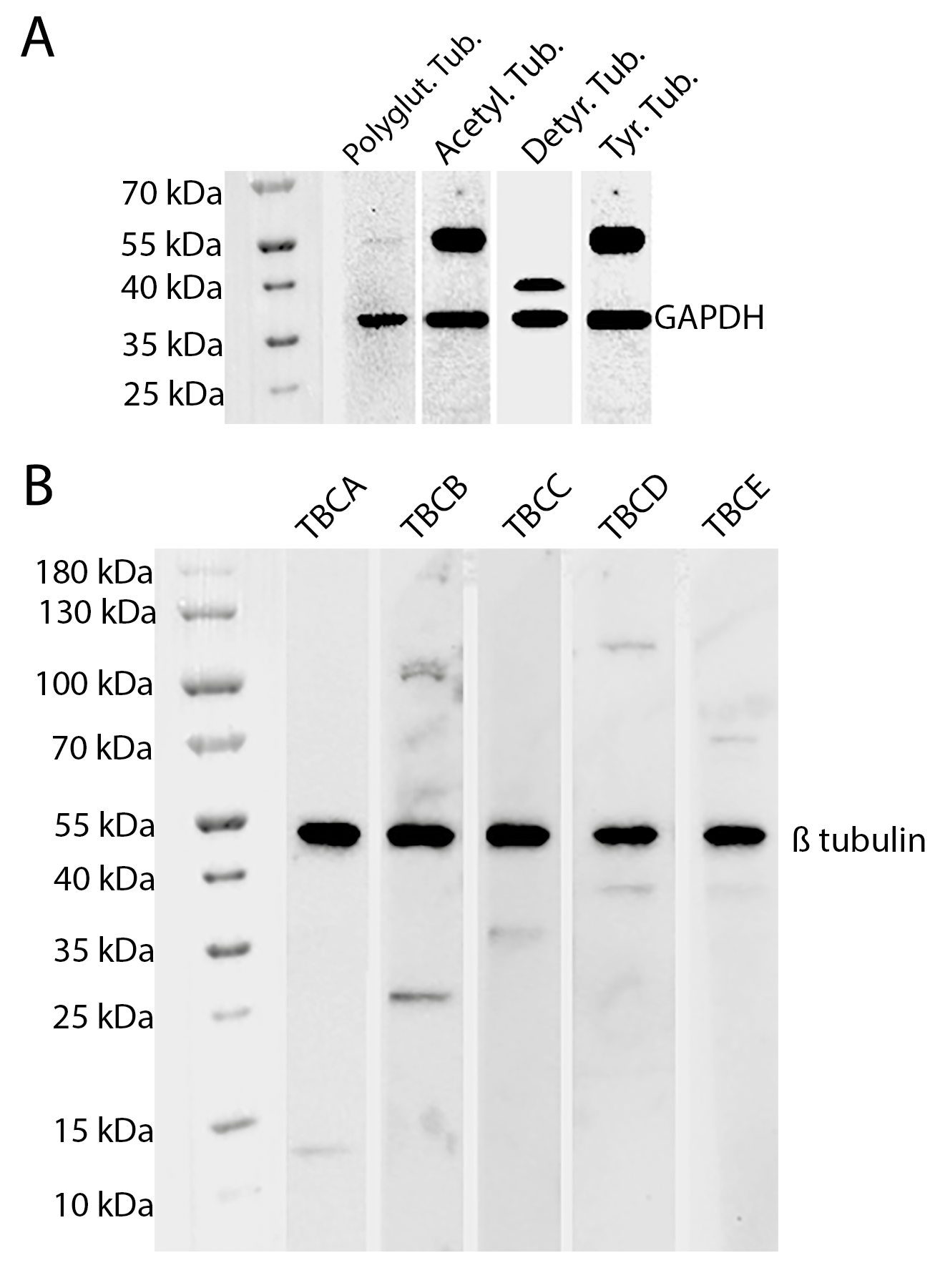

Supplement: Supplementary file 2 — Supplementary file2 Supplementary Figure 2 Antibody validation by Western-blot analysis. Whole-cell lysate of HeLa cell culture was analysed on western blot probed with antibodies against polyglutaminated, acetylated, detyrosinated and tyrosinated PTMs of tubulin and GAPDH as reference protein (a). Antibodies against TBC proteins were investigated and analysed (loading control β-tubulin) (b) (TIF 2841 kb) [file 418_2020_1905_MOESM2_ESM.tif]
